# 4-Methoxymandelic acid: A leatherwood (*Eucryphia lucidia*) honey marker for authentication

**DOI:** 10.1016/j.crfs.2025.101088

**Published:** 2025-05-29

**Authors:** Georgia Moore, Peter Brooks, Asmaa Boufridi

**Affiliations:** a School of Science, Technology and Engineering, University of the Sunshine Coast, Maroochydore BC, Queensland, 4558, Australia; b Centre for Bioinnovation, University of the Sunshine Coast, Maroochydore BC, Queensland, 4558, Australia

**Keywords:** Monofloral leatherwood honey, HPLC-DAD, HPLC-Q-TOF/MS, Phenolic profiles, Phenolic markers, Authentication, 4-Methoxymandelic acid

## Abstract

Phenolic compounds in honey can serve as markers of authenticity for both botanical and geographical origins. Leatherwood (*Eucryphia lucidia*) honey is a uniquely aromatic honey, only produced in Tasmania, Australia. This premium honey contains a distinctive array of phenolic markers useful for chemical fingerprinting. Thirty-two leatherwood honeys were analysed by HPLC-DAD, with samples collected over eight consecutive years and pooled on an annual basis. This study identified the presence of 28 phenolic compounds in leatherwood honey, with 14 compounds classified as potential markers for authentication. Most notably, this study identified, for the first time, 4-methoxymandelic acid using HPLC-Q-TOF/MS as the major phenolic substance in leatherwood honey. Further analysis revealed the presence of 4-methoxymandelic acid in *E. lucidia* nectar and thus a marker for the authentication of leatherwood honey.

## Highlights

• Leatherwood honey samples were collected over 8 years and analysed by DAD-HPLC.
• 14 marker phenolic compounds were identified in leatherwood honey.
• 4-Methoxymandelic acid is unique to leatherwood honey and a marker for authentication.
• 4-Methoxymandelic acid was confirmed in leatherwood flower nectar.

## Graphical abstract

**Figure:**
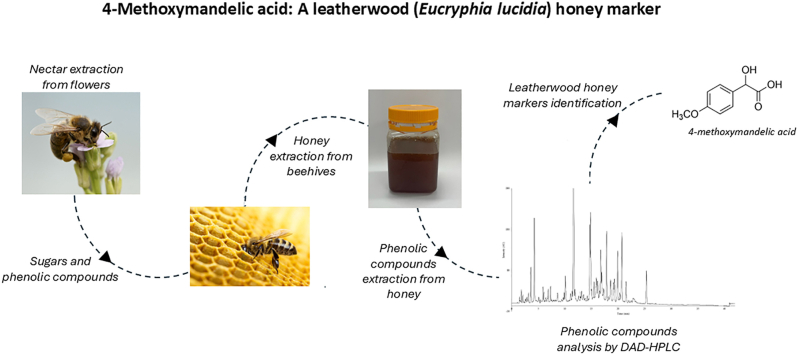

## 1 Introduction

Phenolic compounds, namely phenolic acids and flavonoids, are a natural class of phytochemicals found in honey that confer antibacterial, anti-inflammatory and antioxidant effects (Ayoub et al., 2023). Various studies have demonstrated the relationship between the phenolic content of honey from different floral sources and their antioxidant capability (Jibril et al., 2019; Lawag et al., 2023; Starowicz et al., 2021), with darker coloured honeys having a higher phenolic content and stronger biological properties (Becerril-Sánchez et al., 2021). Phenolic compounds primarily originate from nectar, and despite their relatively minor presence in honey, can be used as markers of botanical origin (Jibril et al., 2019; Cheung et al., 2019).

Many studies have used phenolic compounds to successfully authenticate honey (Farkas et al., 2023; Kędzierska-Matysek et al., 2021; Moore et al., 2025; Nyarko et al., 2023). For instance, methyl syringate and p-hydroxybenzoic acid are proposed markers of rapeseed and buckwheat honey respectively (Kuś et al., 2014), whilst taxifolin is a marker of milkweed honey (Farkas et al., 2023). Similarly, Kato et al. (2012) and Brooks et al. (2024) identified a single marker compound-leptosperin- detected in New Zealand and Australian manuka honeys. Furthermore, Bong et al. (2018) confirmed the differentiation of New Zealand manuka (*Leptospermum scoparium*) and kanuka (*Kunzea ericoides*) honey by comparing their phenolic profiles. Leptosperin, lepteridine, 2-methoxybenzoic acid and 2′-methoxyacetophenone were reported as exclusive to manuka honey whilst lumichrome was characteristic of kanuka honey (Bong et al. (2018)). These studies elucidated floral source by evaluating the presence or absence of one or more specific phenolic compounds.

Nevertheless, it is important to note that the chemical composition can be influenced by factors other than floral source, including geographical location, climate fluctuation, honey age and storage environment (Cheung et al., 2019; Hossen et al., 2017). Consequently, the development of identification criteria for authentication and quality assessment of honey is challenging due to the great variation in honey composition. As honey bees (*Apis mellifera*) cannot be restricted to one floral species in a natural environment, the term monofloral honey describes the predominant nectar source (≥50 %) (Bong et al. (2018); Gheldof et al., 2002; Irish et al., 2011). Foraging honey bees have been observed to collect nectar and pollen up to 6 km from the hive (Visscher and Seeley, 1982). To produce a monofloral variety, apiarists position hives in locations where the nectar flow of interest is in plentiful supply, limiting distractions from other plant species (Yao et al., 2004). Despite this, nectar varieties may become mixed in the hive by worker bees or contaminated during honey extraction further complicating phenolic profiling and honey authentication.

Australia's rich and diverse native flora makes it a leading producer and exporter of unique, high-quality honey products (Irish et al., 2011; Ayton et al., 2019). Specifically, this study focuses on leatherwood honey, which is derived from *E. lucida*, a tree endemic to the highly biodiverse forests of Western and Southern Tasmania (Garland et al., 2022). In addition to producing the uniquely aromatic leatherwood honey, the nectar of *E. lucida* is critical to the sustainability of commercial beekeeping in Tasmania. This essential nectar source ensures a reliable and abundant flow, supporting bees through the winter months (Garland et al., 2022). Given the unique nature of this floral source, distinguishing the phenolic profile of leatherwood honey and identifying its characteristic markers are of great interest to both producers and consumers. Leatherwood honeys have been shown to have a high level of antioxidant activity, up to 7.25 μmol/g Tolox equivalents per gram of honey (Meloncelli, 2019). More recently, the total phenolic content of leatherwood honey (∼58.0 GAE/[mg/100g]), expressed as milligrams of gallic acid equivalent (GAE) per 100g of honey, was comparable to other antioxidant rich honeys including red bell (∼59.4 GAE/[mg/100g]) and jarrah honey (∼50.6 GAE/[mg/100g]) (Lawag et al., 2023; Garland et al., 2022).

Although leatherwood honey harvests were reduced in 2018–2019 due to bushfires, it still accounted for 174 tonnes (43 %) of Tasmania's total honey production, with an estimated value of $2.5 million (AUD) (AgriGrowth Tasmania Tasmanian Beekeeping Survey, 2019 Report, 2019). In 2019, the price of leatherwood honey ($14.45/kg) (Pappalardo et al., 2016) was far greater than farmgate value of Australian honey ($6.50/kg) reported by Clarke et al. (Clarke and Feuvre, 2021). The price of leatherwood honey reflects its status as a high-quality product and popularity with consumers. Therefore, identifying specific chemical markers for leatherwood honey is essential for quality assessment and determining provenance. The objectives of this study were to establish a phenolic profile for monofloral leatherwood honey by high-performance liquid chromatography with diode-array detection (HPLC-DAD) and to identify reliable chemical markers to authenticate all leatherwood honeys, regardless of season or collection variations.

## 2 Results and discussion

### 2.1 Identification of phenolic compounds in leatherwood honey

**Fig. 1:**
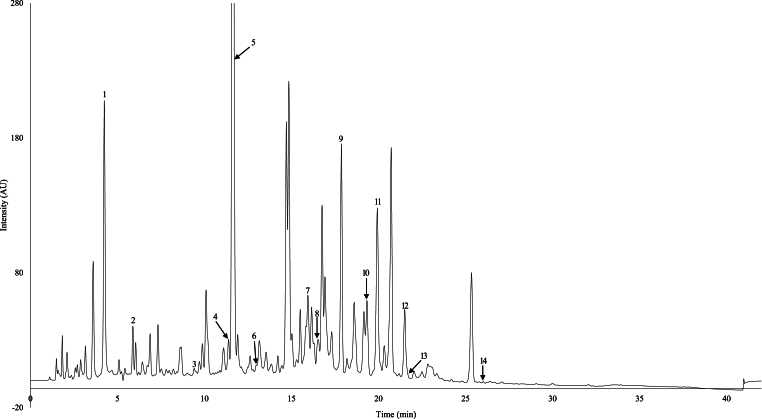
HPLC-DAD chromatogram of sample ‘Combined 2017’ at 220 nm, identifying the 14 marker compounds detected in all leatherwood honeys. Peak labels in Fig. 1 correspond to :1) Kojic acid, 2) Gallic acid, 3) DL-p-hydroxyphenyllactic acid, 4) p-hydroxybenzoic acid, 5) 4-methoxymandelic acid 6) Vanillic acid, 7) 4-Methoxyphenyllactic acid, 8) Ellagic acid, 9) Lumichrome, 10) Genistin, 11) Methyl syringate, 12) Abscisic acid, 13) p-anisaldehyde and 14) Kaempferol.

Tasmanian leatherwood honeys were analysed to identify a range of phenolic compounds and establish authenticity markers. A phenolic compound was considered a floral marker only if it was present in all leatherwood samples. This study utilised a phenolic library of 110 compounds with 28 of these compounds detected in leatherwood honey. Phenolic compounds were quantified using a multiwavelength approach, targeting the most sensitive and selective wavelengths for each compound (Moore et al., 2025). All honey samples shared a very similar phenolic profile, characterised by the presence of thirteen known compounds and one major unknown compound (Fig. 1). Whilst 28 compounds were identified in leatherwood honey, only 14 were quantifiable in all eight samples and considered as marker compounds (Table 1 and Supplementary material- Table S1).

**Table 1:** Concentrated values (mg/kg) of phenolic compounds identified in monofloral leatherwood honey samples (combined 2016- combined 2023). ND – phenolic compound was not detected; (∗)- phenolic compound was detected but not quantified.

	Combined 2016	Combined 2017	Combined 2018	Combined 2019	Combined 2020	Combined 2021	Combined 2022	Combined 2023
**Dihydroxyacetone (DHA)/Methylglyoxal (MGO) (mg/kg)**	DHA = 5 MGO = 9	DHA = 4 MGO = 8	DHA = 7 MGO = 20	DHA = 83 MGO = 40	DHA = 11 MGO = 17	DHA = 2 MGO = 7	DHA = 83 MGO = 30	DHA = 6 MGO = 9
**Phenolic compound (mg/kg)**								
Kojic acid	3.3	10.9	3.2	7.0	8.2	6.1	6.0	3.4
Gallic acid	0.56	0.87	0.51	2.9	0.41	0.53	0.49	1.1
3,4-Dihydroxybenzoic acid	ND	ND	ND	ND	ND	0.29	ND	ND
DL-p-Hydroxyphenyllactic acid	0.36	0.41	0.45	2.4	0.43	0.29	0.65	0.60
Epigallocatechin	ND	ND	ND	15.1	ND	ND	ND	ND
Lepteridine	0.30	0.10	0.44	∗	0.21	ND	0.57	0.29
p-Hydroxybenzoic acid	1.5	1.9	1.6	2.5	1.7	1.6	1.9	2.0
4-Methoxymandelic acid	50.1	84.0	50.7	76.2	53.4	60.9	67.6	115.3
Leptosperin	ND	ND	ND	7.9	ND	ND	6.5	2.6
Vanillic acid	0.17	0.13	0.26	0.13	0.19	0.10	0.11	0.15
Syringic acid	0.61	ND	ND	ND	0.60	ND	0.57	0.77
3-Phenyllactic acid	ND	ND	ND	172.7	ND	ND	ND	ND
4-methoxyphenyllactic acid	6.6	5.5	14.7	51.4	9.8	2.0	19.1	13.3
2-Methoxybenzoic acid	ND	ND	ND	∗	ND	ND	ND	ND
Ellagic acid	0.52	1.57	0.56	7.4	0.51	0.81	0.92	4.1
Taxifolin	0.19	0.71	0.21	2.3	ND	0.27	0.30	1.8
Lumichrome	5.8	6.4	5.9	6.3	6.2	5.8	6.0	7.3
Salicylic acid	ND	0.44	0.53	ND	0.80	0.54	ND	0.33
Genistin	3.9	6.5	7.1	6.0	5.4	6.1	6.0	9.2
Methyl Syringate	8.3	5.1	9.5	57.6	3.4	5.1	10.3	5.3
Abscisic acid	4.3	4.5	4.6	3.1	4.4	5.6	4.8	6.2
p-Anisaldehyde	0.14	0.19	0.14	0.13	0.17	0.17	0.19	0.27
Trans cinnamic acid	ND	ND	ND	ND	0.87	ND	ND	ND
2-Methoxyacetophenone	ND	ND	ND	∗	ND	ND	∗	ND
Apigenin	∗	0.16	0.19	0.17	0.08	0.15	0.09	0.11
Pinobanksin	ND	ND	ND	ND	ND	ND	∗	ND
Kaempferol	0.11	0.14	0.13	0.38	0.13	0.15	0.14	0.14
Pinocembrin	ND	0.08	ND	0.12	0.29	ND	0.18	0.20

**Fig. 2:**
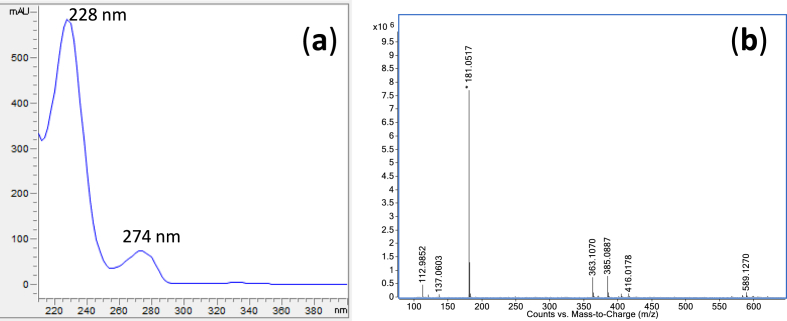
(a) UV spectrum of 4-methoxymandelic acid showing the maximum absorbance at 228 and 274 nm; (b) mass spectrum of 4- methoxymandelic acid.

These consistent markers in leatherwood include (by order of elution): kojic acid, gallic acid, DL-p-hydroxyphenyllactic acid, p-hydroxybenzoic acid, unknown marker 1 (Um1), vanillic acid, 4-methoxyphenyllactic acid, ellagic acid, lumichrome, genistin, methyl syringate, abscisic acid, p-anisaldehyde and kaempferol labelled in Fig. 1. Supplementary Table S1 presents the UV spectra of the aforementioned markers. The unknown marker compound was the dominant peak in leatherwood honey with a retention time of 11.63 min, and exhibited a UV profile (Fig. 2a) similar to the 4-methoxyphenyllactic acid skeleton (Supplementary Table S1), showing maximum absorbance at 228 and 274 nm.

### 2.2 Identification of unknown marker 1

**Fig. 3:**
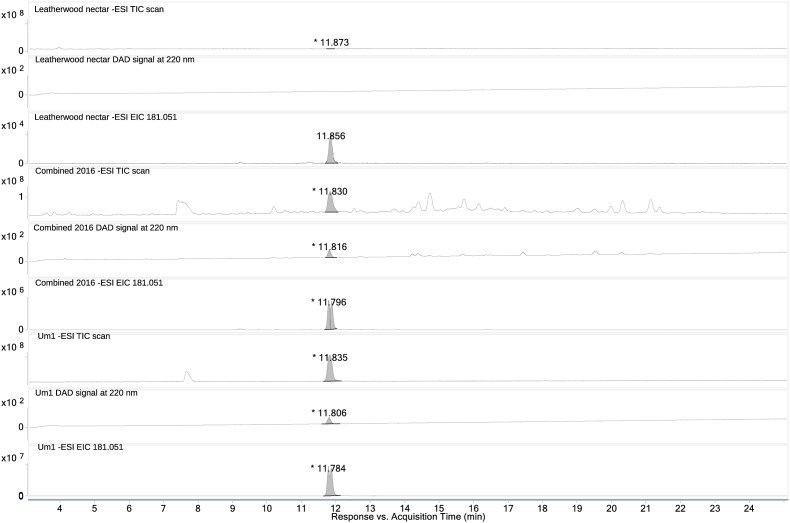
Mass spectrum (TIC scan), DAD signal at 220 nm and an extracted ion chromatogram (EIC) at 181.051 m/z of 4-methoxymandelic acid (Um1) confirmed in leatherwood nectar extract, combined 2016 honey sample extract and analytical standard.

The importance of Um1 as a chemical marker for authenticating leatherwood honey was highlighted in this study. Honey samples were analysed by HPLC- quadrupole time-of-flight mass spectrometer (HPLC-Q-TOF/MS), which confirmed the molecular formulae of Um1 was C_9_H_10_O_4_, with an m/z in negative mode of 181.0515 [M-H]. In 1995, Rowland et al. (1995) identified methyl 2-hydroxy-2-(4-methoxyphenyl) acetate (methyl 4-methoxymandelate) in methylated leatherwood extracts through GC-MS analysis. This previous study suggested that this compound may be present in honey as 2-hydroxy-2-(4-hydroxyphenyl) acetic acid, also known as 4-hydroxymandelic acid. However, our study found that the underivatised form of this compound is present in leatherwood honey as 4-methoxymandelic acid. Confirmation was achieved through comparison of the retention time, UV profile and mass spectrum of Um1 with those of an authentic 4-methoxymandelic acid standard (Fig. 2). Comparing phenolic compounds in both honey sample and the nectar from the suspected floral source is crucial to establishing honey traceability. Nevertheless, phenolic compound analysis is performed more frequently with honey than nectar, as honey is easier to collect. HPLC-Q-TOF/MS nectar analysis of *E. lucida* confirmed 4-methoxymandelic acid as a floral marker of Tasmanian leatherwood honey (Fig. 3).

### 2.3 Leatherwood honey markers

The compound 4-methoxymandelic acid was identified for the first time as a signature marker of leatherwood. Its concentrations ranged from 50.1 to 115.3 mg/kg ($\bar{x}$ = 69.8 ± 22.1 mg/kg), approximately four times higher than any other compound identified in leatherwood, as indicated in Table 1. 4-Methoxymandelic acid was previously reported as a compound found in Tasmanian manuka (*L. scoparium*) (Meloncelli et al., 2015), however the current study confirms 4-methoxymandelic acid in leatherwood nectar and honey. This finding suggests that the presence of 4-methoxymandelic acid in Tasmanian manuka honey was most likely due to contamination from leatherwood nectar, particularly given the lower concentrations observed by Meloncelli et al. (2015) ($\bar{x}$ = 15 ± 9 mg/kg; n = 4). As expected, monofloral leatherwood honey exhibit greater concentrations of 4-methoxymandelic acid than in multifloral leatherwood honey. To support the development of labelling thresholds, further targeted studies are needed to quantify 4-methoxymandelic acid across an extensive range of monofloral and multifloral leatherwood honey samples.

The next major phenolic compounds identified in leatherwood honey were 4-methoxyphenyllactic acid (2.0–51.4 mg/kg) and methyl syringate (3.4–57.6 mg/kg). 4-Methoxyphenyllactic acid has previously been found in manuka (751 ± 295 mg/kg) and kanuka (2365 ± 668 mg/kg) nectars (Bong et al. (2018)). In Australia, the dominant species of kanuka (*K. ambigua*) overlaps both in geographical distribution and flowering season with manuka and leatherwood honey (Meloncelli et al., 2015). Therefore, it is likely that the elevated levels of 4-methoxyphenyllactic acid observed in some of the leatherwood honey samples were a result of the presence of manuka or kanuka nectars (kanuka and manuka nectar were not tested in this study). As a result, 4-methoxyphenyllactic acid cannot be considered a distinguishing marker of leatherwood honey, in the same way 4-methoxymandelic acid can.

Methyl syringate was found in all leatherwood honeys with one sample (combined 2019) containing five times the amount of methyl syringate (57.6 mg/kg) as the next highest sample (10.3 mg/kg). Methyl syringate has been previously detected in manuka (*L. scoparium*) and kanuka (*K. ericoides*) nectars by Bong et al. (2018) and McDonald et al. (2018). Interestingly, combined 2019 was the only leatherwood sample to contain dihydroxyacetone (DHA) (83 mg/kg), methylgloxal (MGO) (40 mg/kg), leptosperin and all four chemical biomarkers (DL-p-hydroxyphenyllactic acid, 3-phenyllactic acid, 2-methoxyacetophenone and 2-methoxybenzoic acid) of manuka as defined by the New Zealand Ministry for Primary Industries (Ministry for Primary Industries Mānuka Science Team, 2017).

The presence of DL-p-hydroxyphenyllactic acid (0.3–2.4 mg/kg) in all leatherwood samples indicates possible incorporation of *Leptospermum* during nectar collection. Leatherwood samples (Combined, 2016–2018; Combined, 2020–2023) contained methyl syringate at much lower concentrations (3.4–10.3 mg/kg) than the combined 2019 sample, suggesting a lower level of floral contamination. Methyl syringate was also detected in leatherwood honeys analysed by Meloncelli et al. (2015) and Garland et al. (2022). Although some of these compounds may be found in other honey types, they still contribute to the distinct chemical fingerprint of leatherwood honey in its entirety.

Similarly, lumichrome (5.8–7.3 mg/kg) was observed in all leatherwood honeys in this study. Reported by Bong et al. (2018) as a kanuka marker, the presence of lumichrome in leatherwood honey is likely a reflection of *Kunzea* nectar as a contaminating floral source. The presence of *Leptospermum* and *Kunzea* nectars in leatherwood honey is mostly unavoidable due to similar flowering periods and geographical proximity. Producing a truly monofloral honey is extremely rare, hence monofloral honey is defined as primarily sourced from the nectar of a single plant species (Lawag et al., 2022). Ellagic acid (0.5–7.4 mg/kg; $\bar{x}$ = 2.0 mg/kg) and vanillic acid (0.1–0.26 mg/kg) were confirmed as present in leatherwood honey. This is consistent with Meloncelli et al. (2015) who detected ellagic acid in leatherwood samples (n = 4) and proposed that ellagic acid is an important distinguishing criteria in leatherwood (detected)/manuka (not detected). Ellagic acid was also detected in all 71 leatherwood honey samples analysed by Garland et al. (2022) but only quantifiable in 68 of 71 leatherwood samples with a mean concentration of 4.8 mg/kg.

Kojic acid (3.2–10.9 mg/kg) and p-hydroxybenzoic acid (1.5–2.5 mg/kg) were found in all leatherwood samples. Kojic acid, a carbohydrate derivative, has been confirmed in a variety of honeys, including: manuka (Lawag et al., 2022; Beitlich et al., 2014; Oelschlaegel et al., 2012), kanuka (Lawag et al., 2022; Beitlich et al., 2014), thistle (Di Petrillo et al., 2018), red bell, coastal peppermint, marri and jarrah (Lawag et al., 2023), brush box, coolibah, grey ironbark , macadamia, mugga ironbark, spotted gum and yapunyah (Moore et al., 2025). Similarly, p-hydroxybenzoic acid was a feature common to all honey types in Moore et al. (2025) and has been identified in rapeseed, buckwheat, linden, acacia, honey dew and many other polyfloral honeys (Kędzierska-Matysek et al., 2021). This suggests p-hydroxybenzoic acid and kojic acid are ubiquitous honey markers and the absence of these markers in honey may have potential to detect fraud.

Gallic acid (0.4–2.9 mg/kg) was present in all leatherwood samples. The presence of gallic acid in leatherwood honey was noted by D'Arcy (D'Arcy, 2005) (n = 2) and in 89 % of leatherwood samples analysed (n = 71) by Garland et al. (2022). p-Anisaldehyde (0.13–0.27 mg/kg) and kaempferol (0.11–0.38 mg/kg) were present in leatherwood honey samples; however, their concentrations were too low to contribute significantly to the overall phenolic content. Nevertheless, their presence was consistent across the eight combined samples. Additionally, genistin (3.9–9.2 mg/kg) and abscisic acid (3.1–6.2 mg/kg) were detected in all leatherwood honeys. Interestingly, genistin has only been previously reported in Acacia (*Robinia pseudoacacia* L.) and Vitex (*Vitex negundo* var. *heterophylla Rehd*.) honeys (Lawag et al., 2022; Shen et al., 2018).

## 3 Materials and methods

### 3.1 Leatherwood honey samples

Blue Hills Honey (Australian Quality Honey Pty Ltd) kindly supplied leatherwood honey (n = 32) and nectar (n = 2) from the West Coast of Tasmania between 2016 and 2023 (Table 2). Four honey samples were provided for each respective year. Individual honey samples were authenticated by HPLC-DAD; however, a combined sample approach to authentication was preferred. Annualised pooled samples better represented the phenolic characteristics of a ‘standard’ leatherwood honey profile compared to individual samples where the chemical profile was subject to seasonal and regional variations. Pooled samples were prepared by mixing equal amounts (∼50 g) of individual samples from each collection year.

**Table 2:** Details of the geographical location and collection date for the 32 Tasmanian monofloral leatherwood honeys and 2 leatherwood nectars analysed.

Individual Sample ID	Combined Sample ID	Extraction Date	Location (Postcode)
1623	Combined 2016	February 12, 2016	7330
1622		February 11, 2016	7321
1624		February 15, 2016	7321
1633		March 7, 2016	7321
1707	Combined 2017	March 1, 2017	7321
1708		March 9, 2017	7321
1706		February 22, 2017	7330
1711		March 15, 2017	7321
1825	Combined 2018	February 8, 2018	7330
1829		February 14, 2018	7321
1838		March 2, 2018	7321
1830		February 15, 2018	7321
1919	Combined 2019	February 28, 2019	7214
1920		March 8, 2019	7321
1918		July 27, 2019	7321
1917		July 26, 2019	7321
2011 (A)	Combined 2020	February 2, 2020	7330
2013		February 27, 2020	7321
2015		March 4, 2020	7321
2011 (B)		February 2, 2020	7330
2116	Combined 2021	March 5, 2021	7330
2119		March 15, 2021	7321
2108		February 19, 2021	7321
2114		March 3, 2021	7321
2205	Combined 2022	February 17, 2022	7330
2214		March 3, 2022	7321
2211		February 28, 2022	7469
2218		March 10, 2022	7321
2318	Combined 2023	March 17, 2023	7330
2313		March 2, 2023	7469
2316		March 9, 2023	7321
2317		March 9, 2023	7321

**Nectar Sample ID**	**Floral Source**	**Collection Date**	**Location (Postcode)**
EC001A	*Eucryphia lucidia*	19/02/24	7330
EC001B	*Eucryphia lucidia*	19/02/24	7330

All honey samples were stored at 4 °C prior to analysis, at which time the samples were left at room temperature (1 h). Given the frequent overlap between *E. lucida* and *L. scoparium* in both geographical distribution and flowering season, all honeys were tested for manuka markers dihydroxyacetone and methylglyoxal using the method of Pappalardo et al. (2016).

### 3.2 Chemicals and reagents

The chemicals and reagents used in this study were: HPLC grade acetonitrile (ACN), LCMS grade acetonitrile, water and formic acid purchased from Merck Australia (Victoria, Australia), and trifluoroacetic acid from Sigma Aldrich (Castle Hill, Australia). Water was purified using a Milli-Q® Direct Water Purification System (18.2 MΩ cm) for HPLC-DAD analysis. O-(2,3,4,5,6-Pentafluorobenzyl)hydroxylamine hydrochloride (PFBHA) was purchased from Amadis Chemical. Phenolic standards (Supplementary material-Table S2) were purchased from Fluka Chemicals (Castle Hill, Australia), Sigma-Aldrich (Castle Hill, Australia), Alfa Aesar (Heysham, UK), MolPort (Rīga, Latvia) and sourced from collaborators (University of Western Australia and Auckland University, NZ).

### 3.3 Standards preparation

Standard solutions (100 μg/mL) were reconstituted in mobile phase A (MPA) for 110 analytical standards (0.004 % trifluoroacetic acid and 2 % acetonitrile in MilliQ water) (Moore et al., 2025).

### 3.4 HPLC instrumentation and conditions

Analysis of the honey extracts were performed according to the method published by Moore et al. (2025). Briefly, a quaternary Agilent 1290 Infinity II HPLC equipped with Phenomenex Synergi Fusion-RP column (150 mm × 4.6 mm, 4 μm particle size) and coupled to an Agilent 1260 Infinity DAD was used. Mobile phases consisted of A: MilliQ water: ACN (98:2 with 0.004 % TFA), and B: 100 % ACN, and the flow rate was set at 1.2 mL/min. Gradient program: 0–1 min at 100 % (MPA); 1–31 min from 100 % to 52 % (MPA); 31–35 min from 52 % to 28 % (MPA); 35–36 min 28 % (MPA); 36–37 min from 28 % to 100 % (MPA); 37–42 min 100 % (MPA). Detection was achieved at 200, 210, 220, 240, 260, 280, 300 and 330 nm.

### 3.5 HPLC-Q-TOF/MS instrumentation and conditions

Honey extracts and nectar extracts were analysed by HPLC-Q-TOF/MS using an Agilent 1290 Infinity II system combined with an Agilent 6546 HPLC/Q-TOF mass spectrometer. Chromatographic separations were performed using the same column and program described above. Mobile phases were slightly modified, consisting of water: ACN (98:2) with 0.05 % formic acid (A) and ACN with 0.08 % formic acid (B). The HPLC-Q-TOF/MS was equipped with a dual AJS ESI source and a negative mode was applied. Spectral data were recorded in the mass range of m/z 100–1000 Da, at a capillary voltage of 2500 V, sheath gas flow of 12 L/min and sheath gas temperature of 350 °C. The MS TOF fragmentor and skimmer were set at 120 and 45 V, respectively.

### 3.6 Phenolic compounds extraction and analysis

#### 3.6.1 Extraction of phenolic compounds in honey

A liquid-liquid honey extraction was carried out as described previously by Moore et al. (2025). Briefly, honey (∼1.0 g) was dissolved using an equivalent volume of MPA and two volumes of ACN. Following centrifugation and freezing at −20 °C, the upper phase was collected and a second ACN extraction was performed on the aqueous phase. The combined extracts were evaporated and redissolved in 0.5 vol of MPA before HPLC analysis.

#### 3.6.2 HPLC-DAD analysis of phenolic compounds in honey

Compounds were identified by comparison of retention times and UV/Vis spectra with phenolic standards according to Moore et al. (2025). Phenolic compounds in honey were quantified at their UV absorption maxima using calibrated standards and reported as milligrams of compound per kilogram of honey (mg/kg).

#### 3.6.3 HPLC-Q-TOF/MS analysis of phenolic compounds in nectar

*E. lucida* nectar (n = 2) was collected based on the methodology ofNorton et al. (2015), whereby 10 μL of Milli-Q water was added to a flower cup and mixed via autopipette. As a washing technique was required for collection, analysis was qualitative not quantitative. The collected nectar was transferred to a HPLC vial, and the process was repeated until each vial contained nectar from 10 flower. Nectars were frozen (−18 °C) until analysis (HPLC-Q-TOF/MS conditions outlined in section 2.5). Nectars were prepared for HPLC-Q-TOF/MS analysis by adding 5 μL of Mobile Phase A (water: ACN: 98:2 with 0.05 % FA) to each nectar vial and a 10 μL aliquot transferred into a HPLC vial.

## 4 Conclusions

This study investigated the phenolic and flavonoid profile of Tasmanian leatherwood honey over 8 consecutive years. Kojic acid, gallic acid, DL-p-hydroxyphenyllactic acid, p-hydroxybenzoic acid, 4-methoxymandelic acid, vanillic acid, 4-methoxyphenyllactic acid, ellagic acid, lumichrome, genistin, methyl syringate, abscisic acid, p-anisaldehyde and kaempferol were identified and quantified in leatherwood honey. Low levels of floral contamination by manuka and kanuka were evident and unavoidable in leatherwood samples. This highlights the challenge of establishing authentication criteria for honey as a natural product. Critically, this study identified 4-methoxymandelic acid (Um1) for the first time as the principal compound in leatherwood honey. Furthermore, 4-methoxymandelic acid is derived from the nectar of *E. lucidia* demonstrating plant origin. Hence a combination of 4-methoxymandelic acid with the 13 phenolic compounds, as secondary markers, is necessary to authenticate Leatherwood honey.

## CRediT authorship contribution statement

**Georgia Moore:** Investigation, Data curation, Writing – original draft, Writing – review & editing, Supervision, All authors have read and agreed to the published version of the manuscript. **Peter Brooks:** Conceptualization, Methodology, Investigation, Data curation, Supervision, All authors have read and agreed to the published version of the manuscript. **Asmaa Boufridi:** Conceptualization, Methodology, Investigation, Data curation, Writing – original draft, Supervision, All authors have read and agreed to the published version of the manuscript.

## Data availability statement

Data will be made available on request.

## Funding

This research did not receive any specific grant from funding agencies in the public, commercial, or not-for-profit sectors.

## Declaration of competing interest

The authors declare that they have no known competing financial interests or personal relationships that could have appeared to influence the work reported in this paper.

## Figures and Tables

**Table:** The following abbreviations are used in this manuscript:

ACN	Acetonitrile
TFA	Trifluoroacetic acid
MQ	Milli-Q water
MPA	Mobile phase A
MPB	Mobile phase B
DAD	Diode Array Detection
Q-TOF/MS	Quadrupole time-of-flight mass spectrometer
HPLC	High Performance Liquid Chromatography

## Data Availability

Data will be made available on request.
